# 11β-HSD Types 1 and 2 in the Songbird Brain

**DOI:** 10.3389/fendo.2018.00086

**Published:** 2018-03-12

**Authors:** Michelle A. Rensel, Jessica A. Ding, Devaleena S. Pradhan, Barney A. Schlinger

**Affiliations:** ^1^The Institute for Society and Genetics, University of California, Los Angeles, Los Angeles, CA, United States; ^2^Laboratory of Neuroendocrinology, University of California, Los Angeles, Los Angeles, CA, United States; ^3^Department of Integrative Biology and Physiology, University of California, Los Angeles, Los Angeles, CA, United States; ^4^Department of Ecology and Evolutionary Biology, University of California, Los Angeles, Los Angeles, CA, United States

**Keywords:** glucocorticoid, mineralocorticoid, 11 beta-hydroxysteroid dehydrogenase, stress response, songbird

## Abstract

Glucocorticoid (GC) hormones act on the brain to regulate diverse functions, from behavior and homeostasis to the activity of the hypothalamic–pituitary–adrenal axis. Local regeneration and metabolism of GCs can occur in target tissues through the actions of the 11β-hydroxysteroid dehydrogenases [11 beta-hydroxysteroid dehydrogenase type 1 (11β-HSD1) and 11 beta-hydroxysteroid dehydrogenase type 2 (11β-HSD2), respectively] to regulate access to GC receptors. Songbirds have become especially important model organisms for studies of stress hormone action; however, there has been little focus on neural GC metabolism. Therefore, we tested the hypothesis that 11β-HSD1 and 11β-HSD2 are expressed in GC-sensitive regions of the songbird brain. Localization of 11β-HSD expression in these regions could provide precise temporal and spatial control over GC actions. We quantified GC sensitivity in zebra finch (*Taeniopygia guttata*) brain by measuring glucocorticoid receptor (GR) and mineralocorticoid receptor (MR) expression across six regions, followed by quantification of 11β-HSD1 and 11β-HSD2 expression. We detected GR, MR, and 11β-HSD2 mRNA expression throughout the adult brain. Whereas 11β-HSD1 expression was undetectable in the adult brain, we detected low levels of expression in the brain of developing finches. Across several adult brain regions, expression of 11β-HSD2 covaried with GR and MR, with the exception of the cerebellum and hippocampus. It is possible that receptors in these latter two regions require direct access to systemic GC levels. Overall, these results suggest that 11β-HSD2 expression protects the adult songbird brain by rapid metabolism of GCs in a context and region-specific manner.

## Introduction

Glucocorticoid (GC) hormones regulate numerous biological processes, including crucial actions on the central nervous system such as feedback regulation of adrenal function, activation or suppression of cognitive and locomotor activity, and regulation of feeding behaviors (1). GC secretion is dynamic, varying according to time of day (2, 3), developmental and life history stage (4–7), as well as in response to acute and chronic stress (1). Following synthesis and secretion, primarily from the adrenals, GC effects are regulated by various mechanisms, including binding to circulating globulins that regulate access to tissues and local expression of catabolic and anabolic enzymes in target tissues (8). Ultimately, tissue genomic and cellular responses are guided by the degree to which GC receptors are expressed. Sensitivity to GC effects is mediated through the intracellular mineralocorticoid receptor (MR) and glucocorticoid receptor (GR); acute stress-induced effects are mediated by the lower-affinity GR, while the high-affinity MR mediates baseline and early stress-induced effects (1, 9). Membrane GR and MR, while less studied, are found in both birds and mammals where they likely mediate rapid, non-genomic effects of GCs (10–12).

The 11β hydroxysteroid dehydrogenase enzymes (11β-HSDs) mediate the interconversion of the GC corticosterone (birds, some rodents, and reptiles) or cortisol (other vertebrates; both hereafter referred to as CORT) to an inactive form, 11-dehydrocorticosterone (11-DHC) or cortisone, respectively. The enzyme 11 beta-hydroxysteroid dehydrogenase type 1 (11β-HSD1) catalyzes the conversion of 11-DHC or cortisone into CORT *in vivo* but is capable of catalyzing the reverse reaction *in vitro*; 11 beta-hydroxysteroid dehydrogenase type 2 (11β-HSD2) exclusively inactivates CORT by catalyzing its conversion into 11-DHC or cortisone (13). Dysregulation of these enzymes in peripheral tissues is implicated in mammalian models of hypertension, diabetes, obesity, and metabolic disorder (14). 11β-HSD2 plays a critical role in the mammalian kidney, where its expression preserves aldosterone access to MR, which binds both CORT and aldosterone with equal affinity (15).

In rodents, 11β-HSDs are expressed peripherally and in brain, although their localization and functional significance vary across developmental stages. For example, relatively little 11β-HSD1 is expressed in the early developing rodent brain (15, 16), whereas it is widely expressed in the adult brain and periphery. In the adult brain, this enzyme participates in hypothalamic–pituitary–adrenal (HPA) axis regulation (17, 18) and facilitates GC-induced memory impairments in aging animals (19). Conversely, 11β-HSD2 expression in the adult brain has a limited distribution associated with salt-based aldosterone sensitivity, while 11β-HSD2 is expressed abundantly in the fetal brain and placenta and is hypothesized to protect the fetal nervous system from excess GC exposure (15).

Evidence for 11β-HSD2 expression in the adult human brain is contradictory (20, 21), and it is possible that CORT is metabolized in the human brain *via* a unique hydroxysteroid dehydrogenase (22). Given the documented ill effects of stress and elevated GCs on adult neurogenesis and cognition (23), the presence of region-specific GC-inactivating and regenerating enzymatic machinery in the brain should be adaptive and expected.

Unlike the adult rodent brain, metabolism of CORT *via* 11β-HSD2 has been reported in hatchling and adult songbird brain using chicken-specific PCR primers (24) as well as species-specific primers (25). In the latter study, we confirmed expression of 11β-HSD2 in two regions of the adult zebra finch (*Taeniopygia guttata*) brain, the caudal nidopallium (cNp) and hippocampus (HP). In addition, expression covaried with free CORT sampled in these regions using *in vivo* microdialysis. Specifically, we found that CORT levels were higher in the region expressing lower levels of 11β-HSD2 (25) suggesting that 11β-HSD2 limits bioactive GC exposure in specific regions of the brain.

The cNp and HP both lie adjacent to the lateral ventricular zone (VZ), a region of the songbird brain in which adult neurogenesis is conspicuous and critical for seasonal growth of song control nuclei (26) and recruitment of hippocampal neurons (27). As previous work has established differential effects of GCs on neurogenesis (23), we predicted that both 11β-HSD1 and 11β-HSD2 expression in HP and cNp, as well as another adjacent region, the caudomedial nidopallium (NCM), should provide precise control over GCs within these sensitive proliferative zones. We therefore hypothesized that 11β-HSD2 and/or 11β-HSD1 expression regulates exposure of the adult songbird brain in regions with heightened expression of GC receptors.

We tested this hypothesis in adult male and female zebra finches, assessing expression in six brain regions. We included three brain regions, NCM, cNp, and HP because of their proximity to the VZ, the diencephalon (DIEN), because of its role in regulating HPA negative feedback, and the cerebellum and optic tectum as control regions. Previous studies have described expression profiles of GR and MR in songbirds (28–32). To the best of our knowledge, 11β-HSD1 expression has not been reported in songbirds, with little data on 11β-HSD2. To assess how metabolic enzymes might participate in controlling GC access to its receptors, we used quantitative PCR to simultaneously characterize expression of 11β-HSD1 and 11β-HSD2, as well as GR and MR, in adult brain. While we detected 11β-HSD2 in all brain regions, 11β-HSD1 expression was undetectable. To verify this result, which differs significantly from mammalian patterns of expression (15), we confirmed 11β-HSD1 expression in the brains of developing finches as well as two additional target tissues, the liver and kidney (33).

## Animals and Methods

### Animals

This study was conducted at the University of California, Los Angeles. All procedures involving animals were approved by the Chancellor’s Animal Research Committee. We utilized adult (>100 days of age) non-breeding zebra finches obtained from our captive colony. For one study, we utilized developing finches of variable ages (see below). Finches in our colony are kept in large open flight aviaries with up to 40 same-sex individuals residing in each enclosure. Breeding cages are comprised of four to five breeding pairs with access to breeding boxes filled with nesting material. Lights are maintained on a 14 h light/10 h dark cycle, and finches are supplied with *ad libitum* seed, water, cuttle bone, and grit at all times. Egg mix and nutritional supplements are provided at least once per week.

### Dissections

**Figure 1:**
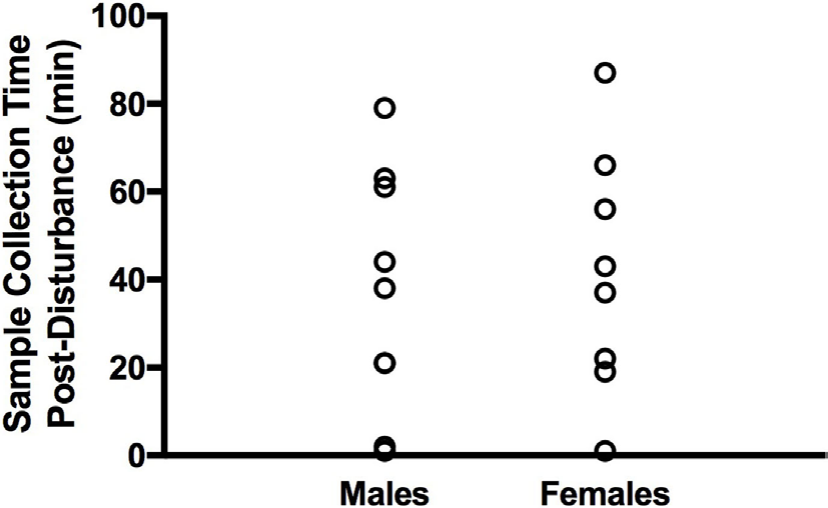
Distribution of tissue collection times post-disturbance in adult male and female zebra finches. Males and females were sampled at similar times.

Groups of four to five adult finches were captured together and transported in a darkened cage to a procedure room where each bird was then processed. Following rapid decapitation, the brain was removed and placed on a Petri dish situated in wet ice (see below). Upon dissection, the six brain regions of interest were immediately frozen on dry ice, transferred to tubes, and kept at −80°C until RNA extraction. The amount of time that passed from initial capture to sacrifice ranged from 1 to 87 min (mean time = 39 ± 6.5 min), and sacrifice time post-capture was included as a covariate in statistical analyses. Sampling times and ranges were highly similar between males and females (Figure 1). A total of 18 birds were sacrificed in this manner, over the course of four sessions in 2 days (one AM and one PM session/day—the order of sexes captured was counterbalanced across days). The same aviary was never entered more than once per day to reduce potential stress effects on gene expression.

Brain regions were dissected as follows: with the brain ventral side down, the whole cerebellum was removed. Next, we made two parasagittal cuts ~1 mm from the midline from the caudal to the rostral end, then removed 5 mm of the rostral portion of the brain. The HP was then carefully separated from the caudal portion and removed bilaterally [see Ref. (25)]. Using watchmaker’s forceps, we next collected a roughly 1 mm^2^ region of the underlying telencephalon (TEL) containing the caudomedial nidopallium (NCM) [see Ref. (34)]. A 1 mm^2^ portion of cNp located lateral to the position of the HP cut was next made bilaterally [see Ref. (25)]. After removal of the remainder of the TEL, the optic tecta were easily separated. Finally, excess optic nerve and myelin was removed and discarded from the ventral DIEN. Liver and kidney were collected next and frozen.

To compare 11β-HSD1 expression across developmental stages, brain tissue was collected from four individuals per age group: 5–7 days post-hatch (“hatchlings”), 25 days post-hatch (“fledglings”), and 75 days post-hatch (“juveniles”). The entire TEL (including underlying DIEN) was collected from hatchlings due to their very small size, while only the caudal TEL was utilized from fledglings and juveniles. The HP was removed from the caudal TEL of these latter ages (for other studies). To enable a direct comparison of expression across ages, the remaining caudal TEL from four of the adults utilized in the main study above was processed in parallel with these developing finch samples.

### RNA Extraction and cDNA Synthesis

RNA was extracted from frozen tissue samples using the Trizol method according to the manufacturer’s guidelines (Ambion). All centrifugation steps were performed at 4°C. Briefly, tissue was homogenized in 1 ml cold Trizol for a maximum of 45 s, followed by centrifugation at 12,000 *g* for 10 min. The supernatant was decanted and incubated at room temperature for 5 min, and 200 µl of chloroform was then added. Samples were vigorously shaken, incubated at room temp for 3 min and then centrifuged at 12,000 *g* for 15 min. After centrifugation, the aqueous layer was carefully removed and placed in a new tube. Next, 500 µl of isopropanol was added to each tube. For small tissues that produced tiny RNA pellets (HP, NCM, and cNp), we added 1 µl of 15 mg/ml glycoblue (Invitrogen) to improve visibility. Tubes were briefly vortexed, incubated at room temperature for 10 min, centrifuged at 12,000 *g* for 10 min, and the supernatant removed, leaving the RNA pellet in place. Finally, 1 ml of 75% ethanol (4°C) was added to the tube, and the tube was inverted to ensure that the pellet was free-floating. After centrifugation at 7,500 *g* for 5 min, the ethanol was carefully pipetted out of the tube, and residual ethanol was allowed to evaporate. The RNA pellet was then resuspended in 10–120 µl of sterile water based on pellet size, vortexed and then heated in a water bath at 58°C for 10 min. RNA quantity and integrity was then determined *via* nanodrop. Total RNA concentrations ranged from 100 to 600 ng/µl, and *A*_260/280_ ratios were between 1.8 and 2.15.

To prepare cDNA from RNA samples, 600 ng RNA was reverse transcribed. Briefly, 0.5 µl of DNAse (Promega) and 1.1 µl of DNase buffer were added to each sample, then incubated at 37°C for 30 min and 65°C for 10 min. Next, 1.5 µl of oligoDT (Sigma) and 0.5 µl dNTPs (Bioline) were added, and tubes were incubated for 10 min at 65°C. Finally, a mix of reverse transcriptase (Superscript II, Invitrogen; 1 μl/sample), RT buffer (4 μl/sample), DTT (1 μl/sample), and RNAse inhibitor (RNAsin, Promega; 1 μl/sample) was added, followed by incubation at 42°C for 50 min and 70°C for 15 min. Samples were then frozen at −20°C until qPCR analysis.

### Quantitative PCR

Mineralocorticoid receptor, 11β-HSD2, and 11β-HSD1 qPCR primers were designed based on the zebra finch genome using Primer3Plus and PrimerBlast (NCBI). GR primers were taken from Banerjee et al. (35). Concentrations were optimized for each primer pair and are listed along with primer sequences and amplicon lengths in Table 1. We used an Applied Biosystems 7300 Real-Time PCR system to quantify gene expression relative to glyceraldehyde-3-phosphate dehydrogenase (GAPDH) in samples using the SYBR Green method. Samples were run at a 1:10 dilution and in duplicate wells. Reaction volume was 25 µl, and cycling conditions were as follows: (1) 2 min at 50°C, (2) 10 min at 95°C, (3) 15 s at 95°C, (4) 1 min at 60°C, repeat steps 3 and 4 40 times, (5) 15 s at 95°C, (6) 1 min at 60°C, (7) 15 s at 95°C, and (8) 15 s at 60°C.

**Table 1:** Quantitative PCR primer details.

Gene name (accession #)	Primer sequence (5′–3′)	Amplicon length (bp)	[Primer] per reaction (μM)
Mineralocorticoid receptor (NM_001076690)	F: AAGAGTCGGCCAAACATCCTTGTTCTR: AAGAAACGGGTGGTCCTAAAATCCCAG	150	0.3
Glucocorticoid receptor (XM_002192952.3)	F: TGCAGTACTCCTGGATGTTCCR: GAGCATGTGTTTGCATTGTTC	155	0.3
11 Beta-hydroxysteroid dehydrogenase type 2 (XM_002187455.3)	F: AAAACAGGGACAACATGCGAR: CCCCTCTGTGATGCTGTTCA	189	0.6
11 Beta-hydroxysteroid dehydrogenase type 1 (XM_002196384.1^a^)	F: CATCCATAGCGGGTAAAATTGR: CGCTCTCTGTGTTGATGTAGC	162	0.3
Glyceraldehyde-3-phosphate dehydrogenase (NM_001198610.1)	F: TGACCTGCCGTCTGGAAAAR: CCATCAGCAGCAGCCT	70	0.3

*^a^This transcript is “11 beta-dehydrogenase 1-like” in NCBI; Ensembl lists a nearly identical transcript as “HSD11B1.” Therefore, we designed qPCR primers to cover a region of the transcript found in both versions*.

Standard curves were prepared for each gene and plate to confirm reaction efficiency (90–110%) and standard curve linearity (≥98%). For GR and MR, each plate contained a standard curve prepared from a mix of 1:1 cDNA representing all samples from the region represented on the plate. We included positive control tissue in the adult 11β-HSD2 standard curves, pooling all brain regions together with a small amount of kidney cDNA. The adult 11β-HSD1 standard curve was constructed from liver cDNA. All standard curves utilized a fourfold dilution with curves extending from 1:1 to 1:256 for GR, MR, and GAPDH, and standard curves ranging from 1:1 to 1:1,024 (11β-HSD1) or 1:4,096 (11β-HSD2). All sample values fell within the bounds of the standard curve for each gene and plate (except for 11β-HSD1; see below). Preliminary optimizations for 11β-HSD1 indicated low to no expression in adult brain; therefore, all samples within a given brain region and a given sex were pooled for the assay instead of running individual samples (*n* = 12 pooled samples from males and females in six regions).

In the absence of 11β-HSD1 expression in regions of the adult brain examined, we conducted further tests to verify the specificity and accuracy of our 11β-HSD1 primers. We first created individual pools of caudal and whole TEL (all ages), liver (all ages), and kidney (adults) and optimized the qPCR reaction. After confirming amplification in all three pools, we sequenced products and used NCBI Blast to confirm the specificity of the products. Because we successfully amplified 11β-HSD1 in the TEL pool, we next ran a single qPCR plate with TEL samples from all four ages (*n* = 4 per age). We constructed the standard curve for this plate from a pool of TEL, kidney, liver, and adrenal cDNA.

Specificity of amplification for GR, MR, 11β-HSD2, and 11β-HSD1 was established by (a) confirming the presence of a single peak on dissociation curves and (b) sequencing and subsequent BLAST analysis of qPCR products. Additional sequencing and gel electrophoresis were used to confirm expression of 11β-HSD1 in hatchling brain (see Results). No reverse transcriptase (no RT) controls were run for each gene to assess DNA contamination. We also confirmed that reaction mixes were not contaminated by running no-template controls on each plate.

Expression levels were calculated using the delta cycle threshold (CT) method, where expression = 1,000*power [2, ^−(CT gene − CT GAPDH)^]. GAPDH was utilized as a reference gene for this calculation, as expression is relatively stable in the songbird brain (25, 36).

### Statistics

Regional variation in MR, GR, and 11β-HSD2 relative expression was assessed using linear mixed models and general linear models where appropriate. For each gene, brain region, sex, the sex by brain region interaction, and sacrifice time post-disturbance were included as fixed effects, and bird ID (*n* = 17; one female was excluded from analyses due to aberrant GAPDH results) was included as a random factor to control for repeated sampling within individual birds. Initial analyses included the sex by sacrifice time interaction. In the absence of any sex-specific effects, this term was eliminated from the final models. The sample size in some brain regions was less than 17 due to technical error or depletion of sample (see figure legends for sample sizes). For GR and MR, there was no variance due to bird ID in the initial mixed model; therefore, general linear models were utilized (model results were identical with and without bird ID). If the interaction term was non-significant, it was removed and the model was re-run to obtain the final model. Significant effects of brain region or a significant interaction term were analyzed using LSD *post hoc* tests, with a significance value of *P* < 0.05. Liver and kidney expression levels in three to four samples were used as positive controls and were not statistically analyzed in comparison with brain.

We used linear regression to compare mean expression levels of 11β-HSD2 with those of MR and GR across regions. Using the same procedure, we tested whether regional patterns of GR expression predicted patterns of MR expression.

## Results

### Regional Patterns of Gene Expression in Adult Brain

**Figure 2:**
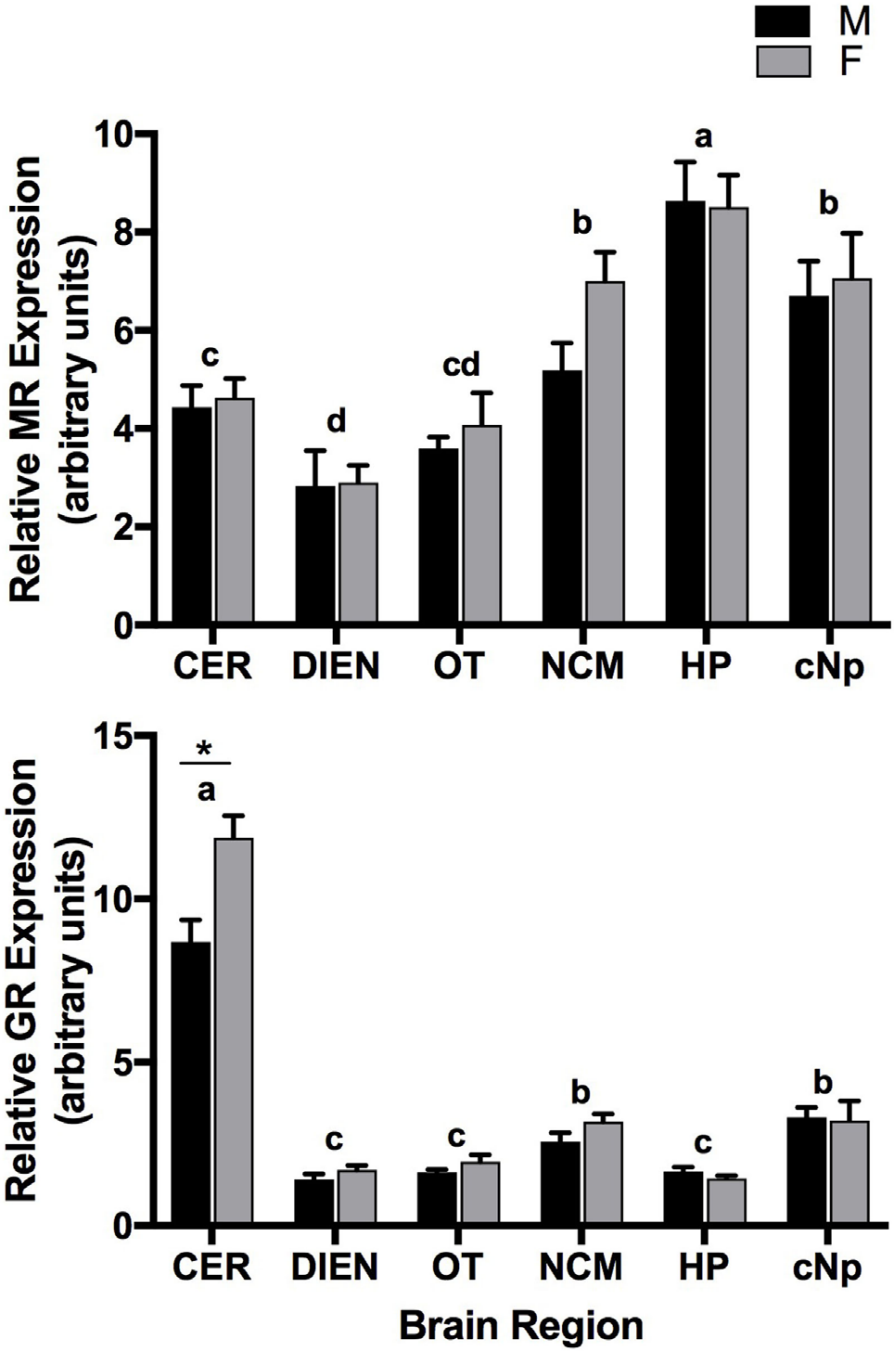
Quantitative PCR results showing mineralocorticoid receptor (MR) (top panel) and glucocorticoid receptor (GR) (bottom panel) expression patterns (relative to glyceraldehyde-3-phosphate dehydrogenase) across six brain regions in adult male (M) and female (F) zebra finches. Letters above bars represent significant differences. *Indicates a significant difference between males and females in CER. Bars are means ± 1 SE. Sample sizes are as follows (*n* = MR/GR): CER, cerebellum (17/16); DIEN, diencephalon (17/16); OT, optic tectum (17/17); NCM, caudomedial nidopallium (16/16); HP, hippocampus (17/17); cNp, caudal nidopallium (13/13).

Mineralocorticoid receptor expression levels differed significantly between brain regions (*F*_5,89_ = 26.2; *P* < 0.001) with highest levels in HP, followed by NCM and cNp, and lowest levels in OT, CER, and DIEN (Figure 2). Levels did not differ between males and females (*F*_1,89_ = 1.9; *P* = 0.171) and were unrelated to time of sacrifice post-capture (*F*_1,89_ = 0.8; *P* = 0.381). There was no interaction between sex and region (*F*_5,84_ = 0.7; *P* = 0.647).

Glucocorticoid receptor expression levels also differed between brain regions (*F*_5,82_ = 191.5; *P* < 0.001) with highest levels in CER, followed by NCM and cNp, and lowest levels in DIEN, OT, and HP (Figure 2). A significant main effect of sex (*F*_1,82_ = 11.4; *P* = 0.001) was driven by the sex*region interaction term (*F*_5,82_ = 6.6, *P* < 0.001). Specifically, GR expression levels were elevated in the CER of females (*P* < 0.001), while there were no sex differences in any other brain regions (all *P* > 0.2). Time of sacrifice post-capture was unrelated to expression (*F*_1,82_ = 0.1; *P* = 0.712).

**Figure 3:**
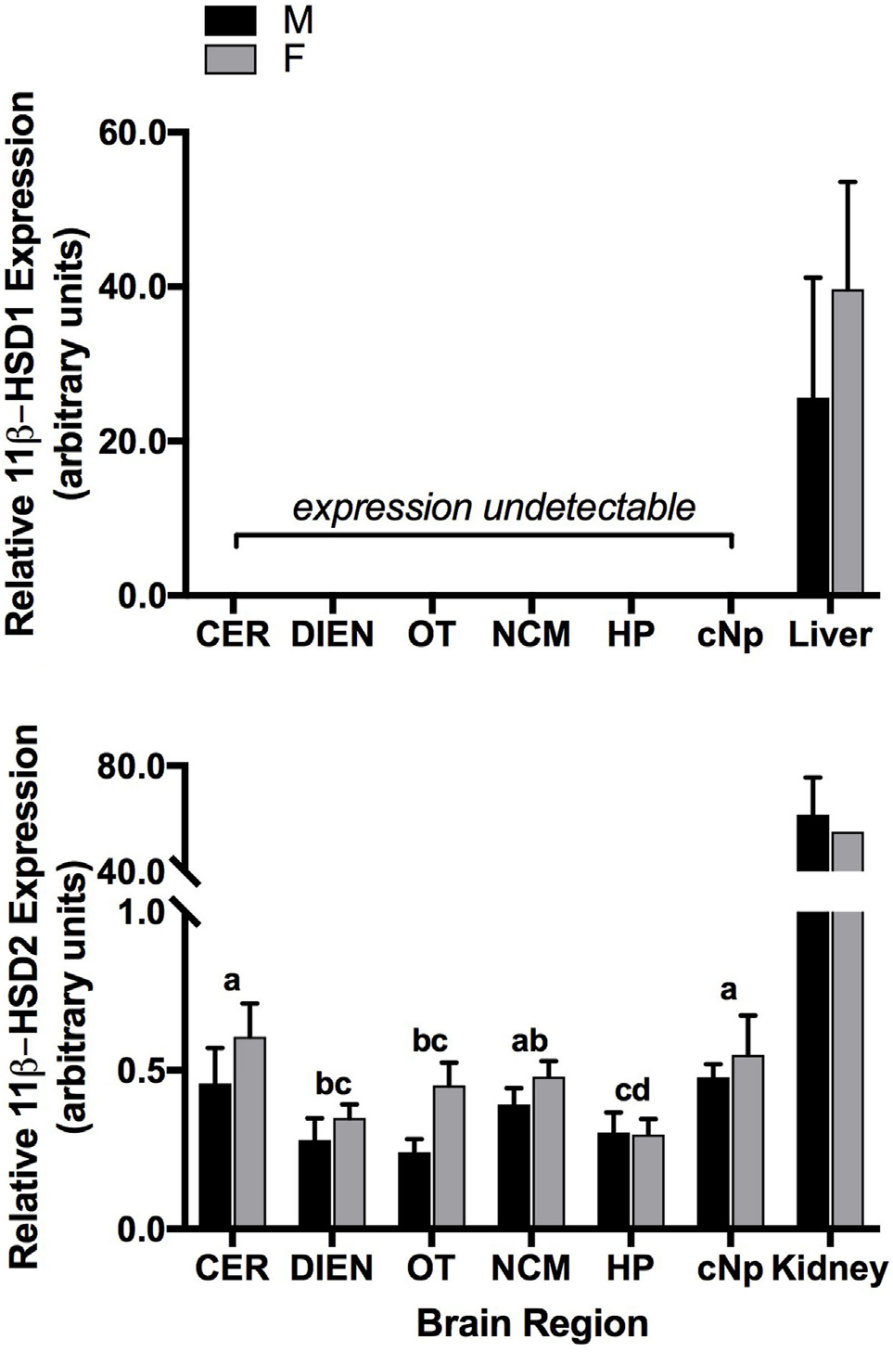
Quantitative PCR results showing 11 beta-hydroxysteroid dehydrogenase type 1 (11β-HSD1) (top panel) and 11 beta-hydroxysteroid dehydrogenase type 2 (11β-HSD2) (bottom panel) expression patterns (relative to glyceraldehyde-3-phosphate dehydrogenase) across six brain regions in adult male (M) and female (F) zebra finches. Letters above bars represent significant differences. Positive control tissues are presented for comparison (11β-HSD1: liver; 11β-HSD2: kidney). Bars are means ± 1 SE. Sample sizes are as follows (*n* = 11β-HSD2): CER, cerebellum (17); DIEN, diencephalon (17); OT, optic tectum (17); NCM, caudomedial nidopallium (16); HP, hippocampus (17); cNp, caudal nidopallium (13).

Whereas expression levels were high in liver, we did not detect 11β-HSD1 expression in brain (Figure 3). Specifically, CT values were undetermined for all brain samples, indicating a lack of amplification within the 40-cycle qPCR program. The 11β-HSD1 primer concentrations (0.6 µM) were determined based on an optimization in liver, as previous validation attempts in brain with differing concentrations of primers and cDNA indicated low to no expression.

In contrast to 11β-HSD1, 11β-HSD2 was expressed in adult kidney and brain and differed significantly among brain regions (*F*_5,89_ = 5.8; *P* < 0.001). Levels were highest in CER, NCM, and cNp, followed by DIEN, OT, and HP (Figure 3). Expression levels did not vary between males and females (*F*_1,89_ = 2.3; *P* = 0.136) or according to time post-capture (*F*_1,89_ = 0.2; *P* = 0.687). There was no interaction between region and sex (*F*_5,84_ = 0.8; *P* = 0.525).

### Coexpression Patterns across Brain Regions

**Figure 4:**
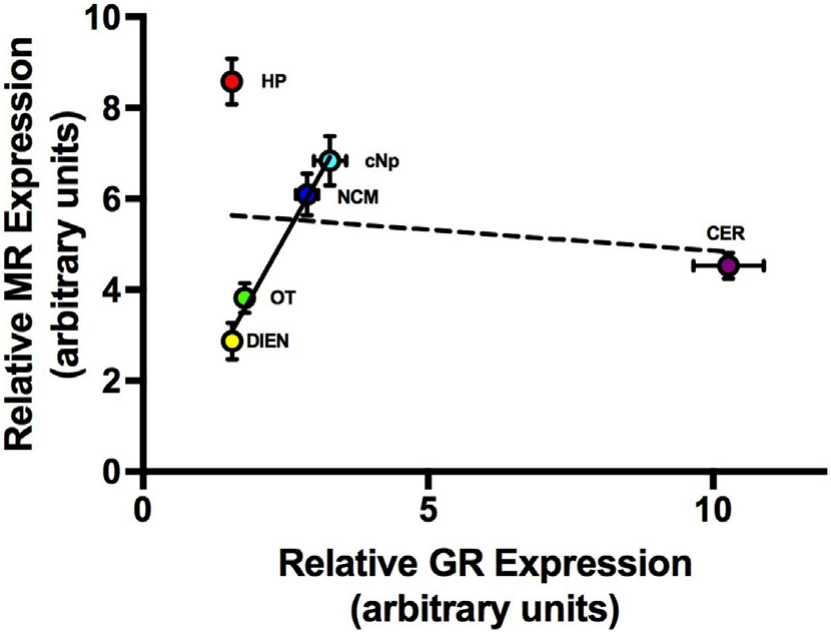
The relationship between average (±1 SE) MR and GR expression levels across six brain regions. Dashed line = best fit line with all six brain regions included. Solid line = best fit line when CER and HP were excluded from the analysis. Abbreviations: CER, cerebellum; DIEN, diencephalon; OT, optic tectum; NCM, caudomedial nidopallium; HP, hippocampus; cNp, caudal nidopallium.

**Figure 5:**
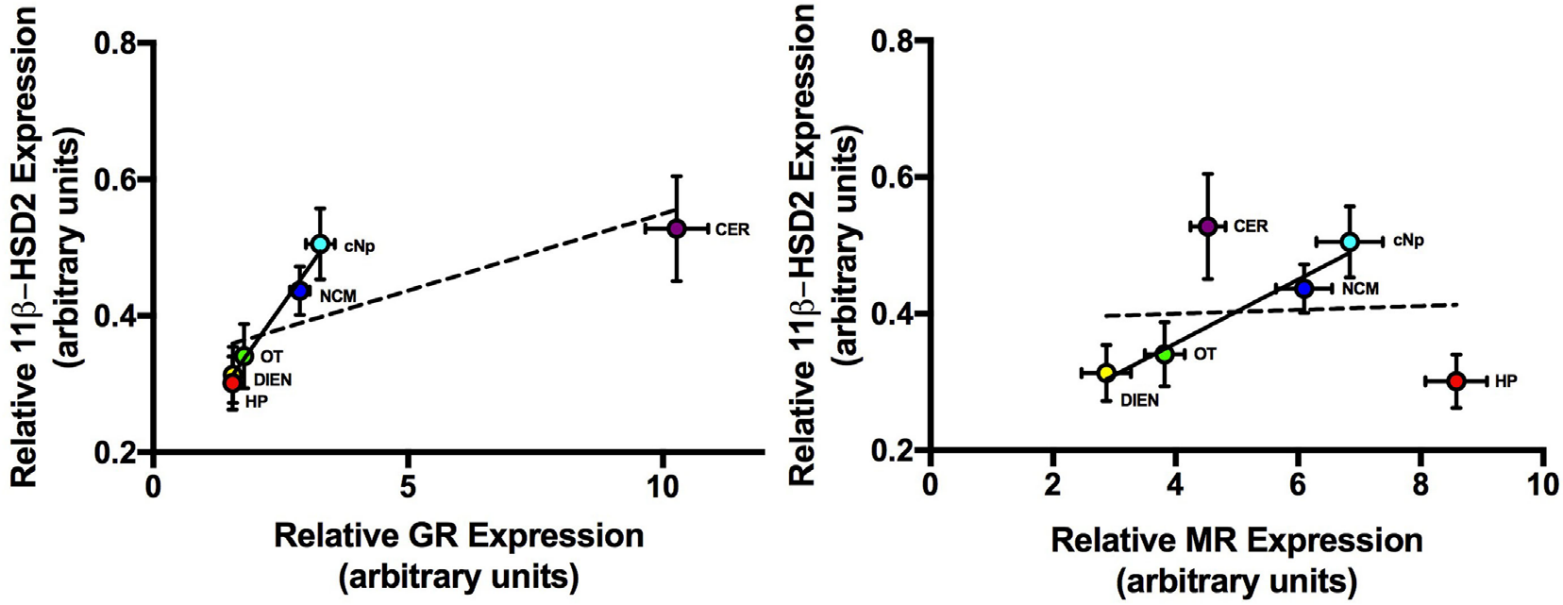
The relationship between average (±1 SE) 11 beta-hydroxysteroid dehydrogenase type 2 (11β-HSD2) and GR (left panel) and average 11β-HSD2 and MR (right panel) expression levels across six brain regions. Dashed line = best fit line with all six brain regions included. Solid line = best fit line when CER and HP were excluded from the analysis. Abbreviations: CER, cerebellum; DIEN, diencephalon; OT, optic tectum; NCM, caudomedial nidopallium; HP, hippocampus; cNp, caudal nidopallium; MR, mineralocorticoid receptor; GR, glucocorticoid receptor.

Mean regional GR expression levels did not correlate with MR (*F*_1,4_ = 0.1; *P* = 0.781). If the two regions with exceptionally high MR and GR were removed, however (HP and CER), there was a strong and significant correlation between GR and MR in the remaining four regions (*F*_1,2_ = 175.7; *P* = 0.006; *R*^2^ = 0.99; Figure 4). Similarly, regional 11β-HSD2 levels were not correlated with GR (*F*_1,4_ = 5.6; *P* = 0.077) or MR levels (*F*_1,4_ = 0.02; *P* = 0.91) when all regions were included in the analyses. However, exclusion of CER and HP resulted in a significant positive correlation between 11β-HSD2 and GR (*F*_1,2_ = 126.1; *P* = 0.008; *R*^2^ = 0.98) and 11β-HSD2 and MR (*F*_1,2_ = 64.5; *P* = 0.015; *R*^2^ = 0.97) among the remaining regions (Figure 5).

### 11β-HSD1 in Developing Zebra Finch Brain and Adult Kidney

After finding that 11β-HSD1 expression was absent in six brain regions of adults, but present in the liver (a major site of expression across taxa), we conducted an additional qPCR optimization to determine whether expression could be detected in a pool of TEL taken from hatchlings, fledglings, juveniles, and adults. We compared the CT value of this pool to those obtained from liver (all ages pooled) and kidney [an additional positive control tissue in rodents (33)]. Liver showed the highest signal, with CT values of ~24, followed by kidney (CTs ~27), followed by TEL (CTs ~30). Lower CT values indicate higher levels of expression in a sample. After sequencing, products from each of these pools correctly BLASTed to the 11β-HSD1 mRNA sequence in NCBI (see Table 1 for details).

To further assess these findings in brain, we used qPCR to examine 11β-HSD1 expression in individual samples from all four age groups. Expression of 11β-HSD1 was detected in 2 of 3 hatchling whole TEL samples (the fourth hatchling sample was contaminated and omitted), 1 of 4 fledgling caudal TEL samples, 0 of 4 juvenile caudal TEL samples, and 0 of 4 adult caudal TEL samples. CT values for the two hatchlings were between 26 and 32, while all other amplification occurred at CT 35 or higher.

We further investigated the presence of 11β-HSD1 expression in these three brain samples by (1) running qPCR products on a gel and (2) sending samples for sequencing. Gel electrophoresis (5 μl product + 1 μl 6× loading dye; 2% gel) on the three brain samples with positive qPCR amplification revealed a single band at the expected size (~162 bp) for both hatchling samples and a faint band for the fledgling sample. Sequenced qPCR products from these three samples matched the 11β-HSD1 sequence in NCBI (using BLAST).

## Discussion

This study tested the hypothesis that the enzymes 11β-HSD1 and 11β-HSD2 are expressed in regions of the adult zebra finch brain with elevated sensitivity to GCs. Whereas widespread changes in GC exposure can be initiated by regulation of adrenal synthesis, secretion, and association with binding globulins, local regeneration and metabolism of GCs, respectively, may enable regions requiring precise GC regulation to control access to receptors on a fine scale (8). Our results in the finch vary significantly from expression patterns reported in rodents (15). In particular, we did not detect 11β-HSD1 expression in adult brain, though we confirmed expression of this enzyme in some hatchlings and fledglings. By contrast, 11β-HSD2 was expressed in all adult brain regions examined. In addition, in adult brains we observed distinct regional patterns of GR, MR, and 11β-HSD2 mRNA, with highest GR expression in CER and highest MR expression in HP. Regional 11β-HSD2 patterns were similar but not identical to GR and MR patterns. This suggests that 11β-HSD2 plays a role in regulating both baseline and stress-induced CORT access to receptors, but that the nature of this activity varies according to brain region and context.

### Regional GR and MR Expression Patterns

Differential expression of GR and MR enables the brain to utilize GCs for a diversity of functions. For example, the paraventricular nucleus of the hypothalamus (PVN), which expresses abundant GR in mammals (37–40), is a key site for stress-induced negative feedback on the HPA axis (41). Similarly, the mammalian HP expresses abundant MR and GR (37, 39, 40) and provides feedback regulation of HPA axis activity (42). The nature of central GR and MR expression and function has been well characterized in mammals (40). In the avian brain, our results on the distribution and abundance of GC receptors align with the handful of other studies, showing elevated GR in CER (30, 31, 35, 43) and MR in the HP [(28, 29, 32, 43); but see Ref. (30, 35)]. We add to these studies the description of relatively low levels of both MR and GR in the songbird DIEN, and relatively high levels in NCM and cNp.

The expression of HP MR and, to a lesser extent, GR suggests that the avian HP plays a similar role in HPA axis regulation in birds and mammals. MR activation in the HP by baseline systemic CORT levels maintains basal activity of the HPA axis through inhibitory projections to the hypothalamus (44). In addition, relatively high GR expression in CER, which we report here, has been observed in the granule and Purkinje cell layers of adult mammals (37, 38) and the external granule cell layer of neonatal mammals (45). We also detected higher CER GR expression in females than males, with no differences elsewhere in brain. The functional significance of this heightened CER sensitivity in females remains unknown. Our results from DIEN differ somewhat from previous observations. For example, several studies show a lack of MR expression in the avian hypothalamus (28, 30, 32) and the mammalian PVN (40), whereas others provide evidence for expression (24, 35). We detected relatively low amounts of both GR and MR in zebra finch DIEN, which includes the hypothalamus. These discrepancies likely result from differential sensitivity of qPCR vs *in situ* hybridization procedures, or because our sampling of the hypothalamus included additional diencephalic regions excluded from other studies.

### GR, MR, and 11β-HSD2 Coexpression

Coordinated GC regulation across the brain is necessary for management of the diverse central functions of CORT (44). It is therefore not surprising that we observed a positive correlation between average GR and MR expression levels across several regions, where those with elevated MR also expressed elevated GR. The same pattern was observed when correlating GR or MR expression with 11β-HSD2 expression: regions with elevated GR or MR expression exhibited the highest 11β-HSD2 mRNA levels. This relationship, however, was not identified in all regions, notably the two regions expressing the highest levels of GR (CER) and MR (HP). While 11β-HSD2 expression was relatively elevated in CER, the degree of elevation did not match that of GR expression. In addition, while HP MR was elevated, 11β-HSD2 expression in this region was among the lowest detected. Taken together, these results suggest that the CER and HP may depend on unmodified systemic levels of GCs to initiate appropriate responses on their respective receptors, while other regions may rely on co-regulation by 11β-HSD2, GR, and MR. Future studies should probe the activity and regulation of 11β-HSD2 particularly within the HP and CER, as these remain sites of elevated GC sensitivity.

The HP of adult finches in this study exhibited a greater degree of MR expression relative to GR than other regions examined, while 11β-HSD2 expression remained low. These results may reflect the critical role of HP MR in providing negative feedback on the HPA axis under basal conditions, as MR is virtually absent from the PVN and pituitary, where stress-induced, GR-mediated feedback predominates (46). While 11β-HSD2 preserves MR access for aldosterone in the kidney (47), HP MR is not an aldosterone target, and 11β-HSD2 expression is therefore unnecessary to enable this function. In addition, it is likely that enzymatic metabolism of GCs *via* 11β-HSD2 in HP would be detrimental, as MR binding in this region provides overarching control over the day-to-day activity of the HPA axis. It is therefore not surprising that 11β-HSD2 transcript levels were relatively low in HP.

In contrast to our finding of elevated MR in HP, GR expression was relatively elevated in the CER. Although this relationship has been observed previously in both birds and mammals, its significance is unclear. Very high GR expression relative to other regions has been observed in the neonatal rat cerebellum (48), and exogenous GC treatments can reduce cerebellar volume and initiate long-term cognitive impairments in children (49, 50). The role of GR in the adult CER is less clear, although stress and GC treatments have been shown to impair motor function in adult rats (51). Interestingly, some mood disorders associated with HPA axis dysregulation (52, 53) have been linked to cerebellar atrophy (54). We detected elevated 11β-HSD2 in adult zebra finch CER, although levels were not as high as expected given the correlation between GR and 11β-HSD2 across other brain regions. This suggests that CORT access to GR plays an important role in the avian CER.

High GR expression in the adult CER, which governs motor function, may be partially responsible for rapid, non-genomic changes in activity observed after acute CORT treatment in songbirds. A single dose of GCs administered non-invasively increased perch hopping activity in white-crowned sparrows within 15 min [*Zonotrichia leucophrys gambelii* (55, 56)]. GCs may affect HP-based cognition as well as motor behavior, leading to rapid changes in cache recovery behavior in chickadees within 5 min of administration [*Parus gambeli* (57)]. Rapid, non-genomic GC effects across vertebrate taxa are likely initiated by membrane GC receptor binding (11). A lower-affinity GC membrane receptor has been characterized in house sparrow [*Passer domesticus* (10)] and zebra finch brain (12). In addition, recent work on membrane GC receptors in mammals suggests that a single gene is responsible for both cytosolic and membrane receptors (11, 58). Therefore, it is possible that our characterization of GR and MR included membrane as well as intracellular GC receptor expression.

Although our study revealed expression patterns for several genes in the GC-signaling pathway, a full understanding of the interaction between GC-metabolizing enzymes and receptors requires assessing whether the molecules in these pathways are co-localized within individual neurons or glia. A higher resolution analysis might also be useful. In mammals, for example, 11β-HSD2 has been localized to the endoplasmic reticulum and the nucleus (59). Thus, cells expressing intracellular 11β-HSD2 might avoid effects of GC metabolism by expressing GR or MR receptors on the cell membrane. An investigation of the subcellular localization of 11β-HSD2 in songbird brain, along with a characterization of membrane GR and MR will provide a more conclusive picture of the nature of 11β-HSD2 expression and function in the songbird brain.

### Functional Significance of 11β-HSD2 Expression in Brain

We observed 11β-HSD expression patterns that differ significantly from those in rodents. First, we found that 11β-HSD2 was expressed throughout the adult songbird brain, a finding that differs markedly from the adult rodent brain with its more limited distribution (15). This result builds on previous findings in our lab, in which 11β-HSD2 transcript, as well as dehydrogenase activity, was reported in hatchling and adult zebra finch brain (24, 25). Second, while we found robust expression of 11β-HSD1 in liver, expression was undetected in the adult zebra finch brain, though this enzyme is widely distributed in the brains of adult rodents (15). There are several possible explanations for this result. First, we did not attempt to quantify 11β-HSD1 expression in *all* brain regions of the adult zebra finch. Therefore, it is possible that other regions do express this enzyme. However, we noted an absence of 11β-HSD1 in the HP, a site of prominent expression in the rodent brain, which suggests a fundamental difference between birds and mammals. Another possibility is that differential splicing produces alternate transcripts in the zebra finch brain and liver. Future work will address this possibility by utilizing primers that target different regions of the gene. Finally, it is intriguing that we were able to detect 11β-HSD1 expression in the brains of very young finches. Such a result suggests that this enzyme could be developmentally down regulated. The role for this enzyme in developing brain is currently unknown, but future work should pinpoint the loci of expression in hatchlings and document more precisely its temporal patterns of expression.

Elevated expression of 11β-HSD2 in the developing rodent brain likely protects the growing brain from potential GC damage. This protection lasts through the first few weeks of neonatal life. This is clearly seen in the external granule cell layer of the cerebellum, where 11β-HSD2 reportedly protects neural progenitor cells from CORT-induced apoptosis, cerebellar atrophy, and developmental deficits (45, 60–62). These patterns in the developing mammal brain raise the question of whether 11β-HSD2 plays a similar role in the adult songbird brain, especially in the CER, cNp and NCM, regions where 11β-HSD2 transcript levels were highest. The HP, NCM and cNp are bordered by the VZ, where neurogenesis persists in adult songbirds (63). We found relatively high levels of GR, MR, and 11β-HSD2 expression in NCM and cNp, highlighting the potential importance of appropriate GC signaling in these areas. While adult neurogenesis in mammals is relatively restricted, it is conspicuous and widespread in songbirds and other taxa, including fish, reptiles, and amphibians (64, 65). GCs can impair hippocampal neurogenesis, depending on dose and context (23, 66–68). As 11β-HSD2 protects neurogenesis during mammalian fetal development (60, 69), it is plausible that the enzyme serves a similar function in adult songbirds, and potentially in fish, where 11β-HSD2 is also widely expressed in the adult brain (70).

The results of this study raise an important question: why are neural 11β-HSD1 and 2 expression patterns so different between rodents and songbirds? One possibility is that these taxa-specific patterns arose as a result of differences in HPA axis activity and regulation. For example, adult mice that are similar in body mass to songbirds have higher circulating baseline and stress-induced GCs, as well as higher brain GC levels when compared with zebra finches (71–73) (Rensel, unpublished data). Thus, one could speculate that mice would express higher levels of 11β-HSD2 to limit CORT exposure to the brain and potential damage to CORT-sensitive neural circuits. Obviously, this is not the case; instead, rodents display widespread expression of the GC-regenerating 11β-HSD1. A second possibility is that differences in neural GC metabolism could be accounted for by differences in MR or GR binding affinities in brain between rodents and songbirds. However, these receptors appear to be relatively conserved in their binding affinities in rodents and birds (10, 74). Interestingly, the mammalian and songbird GR respond quite similarly to GC agonists and antagonists, but some traditional MR agonists in mammals do not work well in songbirds (10), suggesting that there may be MR receptor differences which modify GC action on the receptor between species. Finally, this study and others suggest that neural MR is more widely expressed throughout the songbird brain than the rodent brain (10), providing support for the view that differential neural metabolism may exist to regulate GC action on MR instead of or in addition to GR. Thus, more work is needed to determine if GC actions on MR differ across taxa, possibly explaining differential 11β-HSD expression patterns.

Steroid hormone-binding globulins in the circulation also differ between birds and mammals. Mammals possess both corticosterone-binding globulin (CBG) and sex hormone-binding globulin (SHBG), the latter of which binds androgens and estrogens. By contrast, birds appear to lack SHBG, and instead some sex steroids also bind CBG [primarily androgens (75, 76)]. This difference may affect the amount of free CORT accessible to the brain, which in turn could necessitate neural GC regeneration or metabolism. For example, rodent CBG may bind a greater proportion of circulating GCs, necessitating 11β-HSD 1-based regeneration in brain, whereas free CORT may more easily reach the brain of the songbird because a greater portion of circulating GCs is unbound. However, while CBGs are thought to prevent access to target tissues (77), some studies suggest that CBGs are actually necessary for GC delivery to the brain, making any conclusions preliminary at this time (78). In the end, a vital difference between the brains of rodents and songbirds remains the widespread neuroplasticity that persists into adulthood in the songbird brain and which is present to a much smaller degree in rodents (8). Given the capacity for GCs to influence such plasticity, it seems likely that the expression of CORT metabolic enzymes in the adult avian brain is related to this inherent neuroplasticity.

Overall, our results highlight the complexity of GC signaling in the songbird brain. It is likely that 11β-HSD2 protects sensitive neural circuits from GC access to GR and MR, but the timing and localization of this activity undoubtedly depends on region and dose-specific effects. In addition, it is worthwhile to note that quantification of mRNA expression does not necessarily equate to presence of protein, as Medina et al. (79) documented a disparity between GR and MR mRNA and cytosolic protein in house sparrow brain. Rapid modulation of enzyme activity through post-translational modifications is also likely and may provide an additional layer of control over GC metabolism and action in the songbird brain [e.g., Ref. (80–82)]. Future studies will seek to elucidate the functional importance of 11β-HSD2 in those regions in which it is expressed.

## Ethics Statement

This study was carried out in accordance with the recommendations of the UCLA Chancellor’s Animal Research Committee. The protocol was approved by the Chancellor’s Animal Research Committee.

## Author Contributions

MR and BS conceived and designed the study and wrote the manuscript. MR, JD, and DP conducted the experiments. MR analyzed the data. All the authors provided feedback on the manuscript.

## Conflict of Interest Statement

The authors declare that the research was conducted in the absence of any commercial or financial relationships that could be construed as a potential conflict of interest.
